# Functional traits determine heterospecific use of risk‐related social information in forest birds of tropical South‐East Asia

**DOI:** 10.1002/ece3.2545

**Published:** 2016-10-29

**Authors:** Fangyuan Hua, Ding Li Yong, Muhammad Nazri Janra, Liza M. Fitri, Dewi Prawiradilaga, Kathryn E. Sieving

**Affiliations:** ^1^ State Key Laboratory of BioControlCollege of Ecology and Evolution/School of Life SciencesSun Yat‐sen UniversityGuangzhouGuangdongChina; ^2^ Program in Science, Technology, and Environmental PolicyWoodrow Wilson School of Public and International AffairsPrinceton UniversityPrincetonNJUSA; ^3^ Fenner School of Environment and Societythe Australian National UniversityCanberraACTAustralia; ^4^ Biology DepartmentFaculty of Mathematics and Natural ScienceAndalas UniversityPadangWest SumatraIndonesia; ^5^ Indonesian Institute of Technology (LIPI)CibinongWest JavaIndonesia; ^6^ Department of Wildlife Ecology and ConservationCollege of Agriculture and Life SciencesUniversity of FloridaGainesvilleFLUSA

**Keywords:** functional traits, mobbing, predation risk, social information, tropical rainforest

## Abstract

In birds and mammals, mobbing calls constitute an important form of social information that can attract numerous sympatric species to localized mobbing aggregations. While such a response is thought to reduce the future predation risk for responding species, there is surprisingly little empirical evidence to support this hypothesis. One way to test the link between predation risk reduction and mobbing attraction involves testing the relationship between species’ attraction to mobbing calls and the functional traits that define their vulnerability to predation risk. Two important traits known to influence prey vulnerability include relative prey‐to‐predator body size ratio and the overlap in space use between predator and prey; in combination, these measures strongly influence prey accessibility, and therefore their vulnerability, to predators. Here, we combine community surveys with behavioral experiments of a diverse bird assemblage in the lowland rainforest of Sumatra to test whether the functional traits of body mass (representing body size) and foraging height (representing space use) can predict species’ attraction to heterospecific mobbing calls. At four forest sites along a gradient of forest degradation, we characterized the resident bird communities using point count and mist‐netting surveys, and determined the species groups attracted to standardized playbacks of mobbing calls produced by five resident bird species of roughly similar body size and foraging height. We found that (1) a large, diverse subcommunity of bird species was attracted to the mobbing calls and (2) responding species (especially the most vigorous respondents) tended to be (a) small (b) mid‐storey foragers (c) with similar trait values as the species producing the mobbing calls. Our findings from the relatively lesser known bird assemblages of tropical Asia add to the growing evidence for the ubiquity of heterospecific information networks in animal communities, and provide empirical support for the long‐standing hypothesis that predation risk reduction is a major benefit of mobbing information networks.

## 1 Introduction

Across taxa and ecosystems, animals make wide use of social information from both conspecifics and heterospecifics in daily decision making (Danchin, Giraldeau, Valone, & Wagner, 2004; Seppänen, Forsman, Mönkkönen, & Thomson, 2007). Social information is particularly important for prey species as the reduction of uncertainty in decision making regarding daily activities is of extreme value to the avoidance of mortality (Brown, Laundré, & Gurung, 1999; Schmidt, Dall, & van Gils, 2010; Seppänen et al., 2007). In birds and mammals, alarm calls constitute an important form of risk‐related social information widely used by conspecific and heterospecific prey species to inform their antipredator behaviors (Caro, 2005; Hetrick & Sieving, 2012; Schmidt et al., 2010; Templeton & Greene, 2007; Zuberbühler, 2009). In particular, mobbing alarm calls that consist of harsh, easily locatable vocalizations directed at identified predators not posing immediate danger can elicit the approach, inspection, and/or mobbing behaviors of many heterospecific species across a wide range of bird communities (Forsman & Mönkkönen, 2001; Hurd, 1996; Sieving, Contreras, & Maute, 2004). Prey species attraction to heterospecific mobbing calls and their subsequent participation in mobbing aggregations organized around specific target predators are thought to reduce future predation risk from the target predators (Dugatkin & Godin, 1992; Graw & Manser, 2007; Hurd, 1996). Despite the strong theoretical appeal of this hypothesis, however, there is surprisingly little empirical evidence to link heterospecific attraction to mobbing calls with the reduction in predation risk for the responding species (Magrath, Haff, Fallow, & Radford, 2015).

One approach to establish the link between the attraction to heterospecific mobbing calls and predation risk mitigation involves examining the functional traits defining prey vulnerability to predation risk among species that do and do not exhibit attraction responses to heterospecific mobbing calls. If responding to mobbing calls with attraction behaviors (hereafter “responding to”) reduces predation risk, responding species should be vulnerable to similar suites of predators as the species producing mobbing calls, and thus should share with the mobbing call producers similar functional traits that collectively define vulnerability to their shared predators (e.g., Forsman, Thomson, & Seppänen, 2007; Hurd, 1996). In contrast, nonresponding species should differ noticeably from the species producing mobbing calls in terms of these functional traits. Two of the most important functional traits determining prey vulnerability to predation risk are body size and foraging space use (Klecka & Boukal, 2013; Sridhar et al., 2012). The relative body size of prey species in relation to that of predators fundamentally defines the desirability of prey to predators based on the predators’ physical ability to capture and handle prey (i.e., predator–prey body mass allometry; Brose et al., 2006; Cohen, Pimm, Yodzis, & Saldaña, 1993; Templeton, Greene, & Davis, 2005). Prey foraging space use defines their spatial overlap, and thus probability of encounter, with predators when they are active. In forest birds, foraging height represents an important aspect of foraging space use because most bird species are known to associate with certain foraging heights (del Hoyo, Elliott, & Christie, 1992–2013; Pearson, 1971; Walther, 2002).

Here we combine bird community surveys with behavioral manipulations of a forest bird community in the species‐rich tropical rainforests of South‐East Asia to test whether the functional traits of (1) body size and (2) foraging height predict species’ attraction response to heterospecific mobbing calls. At four forest sites spanning a gradient from intact to secondary forest in lowland Sumatra, Indonesia, we conducted intensive point count and mist‐netting surveys to characterize the resident bird communities. We further conducted standardized playback trials using the simultaneous mobbing calls of five common prey bird species inhabiting these forests in combination with the playback of a common predator (an owl species), and characterized the species responding to these playbacks by quantifying their approach behaviors. The five species producing mobbing calls in the playback stimulus are generally similar in body size and foraging height, while the selected owl species is known to be a target of mobbing by these and other prey species. We characterized the distribution of body size (measured by body mass) and foraging height among responding and nonresponding species, and analyzed whether these functional traits predicted species’ tendency to exhibit attraction behaviors to mobbing calls given their presence in the bird communities.

## 2 Materials and Methods

### 2.1 Study site

**Figure 1:**
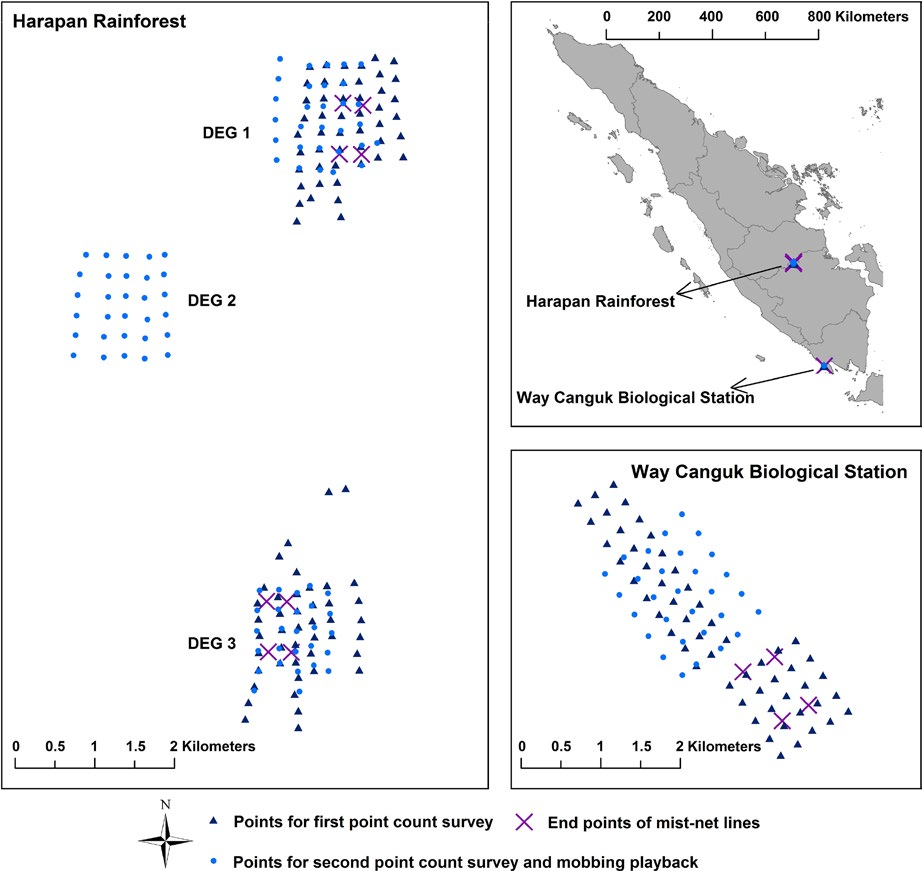
Map of study sites and the locations of sampling points

We conducted fieldwork at four forest sites located within two locations in the rainforests of lowland Sumatra (Figure 1). The Way Canguk Biological Station within the Bukit Barisan Selatan National Park in Lampung Province (5°39′ S, 104°24′ E, 30–60 m a.s.l.) represented an intact primary forest site (hereafter PRIM). The Harapan Rainforest Ecosystem Restoration Site in Jambi Province (2°08′ S, 103°22′ E, 50–80 m a.s.l.) contained three independent study sites of postlogging secondary forest (hereafter DEG1, 2, 3, respectively). The 900‐hectare (ha) Way Canguk Biological Station is probably the last sizable patch of primary lowland rainforest in central or south Sumatra (O'Brien & Kinnaird, 1996), while the Harapan Rainforest Ecosystem Restoration Site encompassed 98,554 ha of secondary lowland rainforest in varying stages of regeneration after selective logging at the time of the study (Hua, Marthy, Lee, & Janra, 2011). The two locations are spaced approximately 600 km away from each other, while the three forest sites within the Harapan Rainforest are spaced ≥2 km apart (Hua & Sieving, 2016).

### 2.2 Study design

We conducted variable‐radius point count (Reynolds, Scott, & Nussbaum, 1980) and mist‐netting surveys of the entire bird community excluding aerial species to characterize the presence/absence status of resident bird species at each study site. Because of their unique strengths in surveying different subsets of the bird community, the two techniques complement each other in providing a thorough characterization of the bird community (Whitman, Hagan, & Brokaw, 1997). By recording the detection distance of each bird individual and thus their natural patterns of proximity to point count stations, the variable‐radius point count also provided baseline data that allowed us to test the validity of potential relationships between species’ functional traits and their attraction response to mobbing calls.

We conducted standardized playbacks of mobbing calls plus owl predator calls along with model presentation of the owl predator to elicit the attraction response from potential prey bird species (Hua & Sieving, 2016). The mobbing playbacks presented naturally occurring, simultaneous mobbing calls of five common, small‐bodied under‐/mid‐storey bird species that occurred at each of our study sites, namely spectacled bulbul *Pycnonotus erythropthalmos*, buff‐vented bulbul *Iole olivacea*, pin‐striped tit‐babbler *Macronus gularis*, dark‐necked tailorbird *Orthotomus atrogularis*, and black‐naped monarch *Hypothymis azurea* (Table S1). The owl playback using territorial calls and model presentation simulated the perched presence of the Sunda scops‐owl *Otus lempiji*, an important predator of the forest mid‐storey on large insects and small vertebrates, including birds, in lowland Sundaland (del Hoyo et al., 1992–2013) and a known target of avian mobbing (FH personal observation). We simulated the presence of the perched owl being mobbed primarily to provide a focal point toward which responding birds targeted their attraction behaviors, and to prolong birds’ behavioral responses during mobbing for better behavioral observation (Chandler & Rose, 1988). It should be noted that because the identity of the predator(s) targeted in the mobbing playback was unknown, the Sunda scops‐owl may or may not present a consistent cue of the target predator with the mobbing calls. However, the territorial call of the Sunda scops‐owl is acoustically much softer than the harsh mobbing calls (i.e., acoustically less dominant through its much slower repetition; Figure S1), and is known to be generally ineffective in eliciting avian attraction responses on its own (Supporting information). We therefore considered that birds attracted to the playbacks were responding first and foremost to the mobbing calls and that the potential inconsistency of predator information between the mobbing and owl playbacks was not a serious problem.

We compiled trait values of body mass and foraging height for all species detected by our community surveys from published sources (Wilman et al., 2014) and analyzed the relationship between these functional traits and species’ attraction response to mobbing calls, combining data from all study sites. We recognize that a critical assumption for our hypothesis is that this relationship arose not as an artifact of the association between species traits and their natural distributional proximity to playback centers (i.e., the tendency of species with certain traits to be distributed close to playback centers leading to an apparently heightened tendency to be attracted to mobbing calls). We addressed this assumption in two ways. First, in counting species as exhibiting attraction responses to the mobbing calls, we did not include the “baseline” bird individuals that were near the playback centers before we initiated playbacks, unless they were subsequently observed actively exhibiting attraction behaviors (e.g., approaching and/or vocalizing toward the playback centers). We recorded baseline bird individuals during a two‐minute interval before each playback trial. Thus, species that were incidentally present near playback centers but were not attracted in were not considered as responding to mobbing calls. Second, we considered the species recorded close to point count stations as a proxy of the species naturally distributed close to playback centers, and analyzed the relationship between their focal functional traits and distributional proximity. A lack of association would suggest that any relationship detected between functional traits and species’ attraction response to mobbing calls was not an artifact of their natural distribution patterns.

### 2.3 Bird community surveys

We conducted bird community surveys at the four study sites between November 2010 and July 2011. With the exception of DEG2, we conducted two rounds of point count survey for each site, respectively, in February–March and June–July of 2011; for DEG2, we conducted one round of point count surveys in June 2011 (Figure 1). We conducted the point counts between 05:50 and 10:30 hr only on days of good weather (i.e., days without rain or strong wind). Point count stations were spaced ≥200 m apart for the first survey round, and ≥250 m apart for the second survey round. Each point count lasted 10 min, during which we recorded all birds seen or heard along with their direction and estimated distance from the point count stations in distance bands of 5, 10, 20, 50, 100, and 150 m. We did not record birds beyond 150 m, and minimized double counting by removing likely overlapping records based on direction and distance estimates. In all, we conducted 151 point counts.

In DEG1, DEG3, and PRIM, we conducted three rounds of mist‐netting surveys, in December of 2010 to January of 2011 and February–March of 2011 at the three sites, and in July (PRIM only) and October (DEG1 and DEG3 only) of 2011; we did not conduct mist‐netting surveys at DEG2 (Figure 1). We conducted mist‐netting at each site in one 450 m × 600 m plot and simultaneously operated 30 mist nets (12 m long, 2.6 m high, and 38 mm mesh size) over three intervals of 2.5 days (totaling 7.5 days of netting effort for each round of mist‐netting). During each interval, net arrays sampled one‐third of the plot before being shifted to cover the next section (Figure S2), and each array consisted of three parallel lines of 10 nets arranged head to tail; net lines were spaced ~150 m apart. We operated nets from 06:00 to 17:30 hr (to 12:00 hr on the third day of each interval) on days without rain or strong wind. We identified, measured, and banded all captured individuals and recorded all recaptures. In all, our mist‐netting effort spanned 783 field hours.

We recorded a species as present at the forest site in question if it was detected by visual observations or auditory cues during point counts or was captured during mist‐netting. We considered a species as naturally distributed close to point count stations if it was recorded within 20 m from point count stations at any time during the surveys, and as naturally distributed far away from point count stations otherwise. We did not use a 15‐m cutoff distance as used in mobbing behavioral observations (see Section 2.4) because we did not use a 15‐m distance band during point counts.

### 2.4 Characterization of the bird community attracted to mobbing calls

We conducted standardized mobbing playbacks in June (DEG 1–3) and July (PRIM) of 2011, between 08:30 and 13:00 hr on days without rain or strong wind. At each study site, we delineated a 125‐ha (1,000 m × 1,250 m) plot with 30 sampling points spaced 250 m apart at which we conducted playbacks of mobbing plus owl calls along with owl model presentation and behavioral observations (Figure 1; Hua & Sieving, 2016). The mobbing playback recording was a 28‐s clip recorded during a naturally occurring mobbing event in the lowland rainforest of northern Sumatra (Lamno, Aceh Province; graciously provided by B. van Balen). The cause of the mobbing event was unclear, but was probably an avian predator (B. van Balen, personal communication). The Sunda scops‐owl territorial recording was a 74‐s clip recorded from West Kalimantan and downloaded from the online repository xeno‐canto.org (van Balen, 2008); it was of the same dialect as that of the Sunda scops‐owls at our field sites in Sumatra (FH personal observation). Sonograms of both recordings are provided in Figure S1. We set both recordings on a 10‐min noninterrupted loop. For model presentation of the owl, we used a wooden model in perched posture (Figure S3).

Within 10 m of each sampling point, we set up the playback system that consisted of (1) the owl model and (2) a camouflaged speaker for the owl vocalization (both on top of a 3‐m‐long pole), and (3) a second camouflaged speaker for mobbing calls in vegetation 1–2 m away from the pole at ~2 m above ground facing the owl model. We connected each speaker via extension cords to an iPod player and controlled them remotely. We considered the pole as the playback center, and demarcated a 15‐m distance radius from the pole in four ordinal directions with colored ribbons to provide visual aid for behavioral data collection. After setting up the playback system, we retreated to 10 m away from the owl model, waited for 2 min to allow any possible disturbance caused by our movement to go away, and conducted playbacks nonstop for 10 min. During the two‐minute preplayback period, we also recorded the identity and number of birds within the 15‐m radius to characterize the “baseline” birds, that is, birds that were naturally near the playback center. We standardized the playbacks at natural and undistorted volumes at a 10‐m distance.

During the 10‐min playbacks, we recorded the presence/absence of species approaching to within 15 m from the playback centers. Therefore, species approaching to within 15 m from the playback centers were considered as exhibiting an attraction response to the mobbing calls. Data on species identity and abundance were collected mainly by one observer (LMF for the three sites at Harapan Rainforest, and another field assistant for the site PRIM), and were supplemented by a second observer (FH) who, while taking behavioral observations on bird individuals on voice recorders, was able to opportunistically record the identity of species and number of individuals. In all, we conducted 101 playback trials.

### 2.5 Data on risk‐related functional traits of bird species

We obtained data on the metrics of body mass (in grams) and foraging height (in meters) for all bird species recorded in our study from the EltonTraits 1.0 data base (Wilman et al., 2014). In particular, we calculated each species’ foraging height based on the information on foraging stratum provided in EltonTraits 1.0, that is, the percentage of time a species spends foraging at each of the “ground,” “understory,” “mid‐high,” “canopy,” and “aerial” strata (percentages for these five strata sum up to 1 in EltonTraits 1.0; Wilman et al., 2014). For this purpose, we presumed the height of these five strata to be respectively 0, 2, 10, 25, and 30 m, values that we considered reasonable for a typical, undisturbed South‐East Asian lowland rainforest, and averaged these heights weighted by their corresponding percentages. To ensure the robustness of our results against these presumed height values, we additionally adopted four alternative, lower sets of height values to represent a range of secondary forest conditions (Table S3). Because results were qualitatively consistent across sets of height values (Tables S4–S9), below we only present results based on the main set described above (i.e., 0, 2, 10, 25, and 30 m for the five strata).

### 2.6 Statistical analysis

Of all species detected by our surveys, we retained only resident, nonraptorial species (excluding owls, hawks, and falcons; i.e., only prey species) for analysis because we conducted mobbing playbacks during the avian breeding season of the Northern Hemisphere when winter migrants such as some leaf warblers and flycatchers were not present. We also removed from analysis the five species represented in the mobbing playback file to focus our analysis only on the heterospecific use of social information. We measured the “tendency to exhibit attraction behaviors to mobbing calls” at the level of study sites and for each species. For a given study site and species, we defined this tendency as the probability of the species being attracted to mobbing calls during at least one playback trial (measured by its presence/absence within 15 m from the playback center) given its presence in the bird community at the site (measured by its detection/nondetection in the combined survey data sets from point counts and mist‐netting). Similarly, we defined the tendency to be distributed close to point count stations at the level of study sites and for each species, as the probability of the species being recorded within 20 m from a point count station at least once given its presence in the bird community at the site.

To characterize the distributions of body mass and foraging height of bird species, we plotted the kernel density curves of these two functional traits among responding and nonresponding species and displayed the trait positions of the five species producing the mobbing calls. To test how these traits predicted species’ tendency to respond to the mobbing calls, we constructed two‐tiered hierarchical models that were based on the maximum‐likelihood estimation approach. We used two complementary trait measures: raw trait values directly extracted from EltonTraits 1.0 data base (Wilman et al., 2014), and relative trait values calculated as the absolute difference between each species’ trait value and the mean trait values of the five species producing the mobbing calls. The test based on relative trait values thus explored how species’ tendency to respond related to their trait similarities with species producing the mobbing calls.

The first tier of the hierarchical models defined a species’ probability, at the level of study sites, of responding to the mobbing calls (given its presence in the community), *p*, as a function of body mass, *m*, and foraging height, *h*. For a given species, we assumed the same *p* for all sites to enable the analysis of trait effects (see Section 4). The second model tier used the binomial distribution with probability *p* obtained from the first tier to define whether a species, given that it was present at a study site, would respond to the mobbing calls. Depending on the number of sites each species was present at, this second model tier constituted a binomial trial with the number of draws ranging from one (if the species occurred at one site) to four (if the species occurred at all four sites); this model tier thus assumed independence among study sites in terms of each species’ attraction status. The two model tiers therefore combined to mathematically describe the number of sites where a species was expected to respond to mobbing calls, out of all the sites at which it was present. We estimated the effect of the covariates in the first‐tier models using the maximum‐likelihood approach.

For hierarchical models using raw trait values, we constructed a full set of candidate models (involving all combinations of covariates based on the global model; see below) for the first model tier and used model selection based on Akaike's information criterion (AIC) to select the best model (i.e., the model with the smallest AIC score; Burnham & Anderson, 2002). Model selection thus only concerned the first model tier. We used model selection involving multiple candidate models because we entertained alternative forms of relationship between the response variable of responding tendency and the explanatory variables of traits. In particular, in the global model, the logit of *p* followed a linear relationship with *m*,* h*, along with their respective quadratic terms (Equation (1)). For hierarchical models using relative trait values, we based our inference on only one candidate model involving a linear relationship between responding tendency and relative trait values (Equation (2)), because our study question implies that species closer in trait space to the species producing the mobbing calls would have higher responding tendencies (e.g., Forsman et al., 2007; Hurd, 1996). We centered and scaled the trait values to facilitate model convergence.

(1) $$\log\left(\frac{p}{1-p}\right)∼m+m^{2}+h+h^{2}\;\text{(for raw trait values)}$$

(2) $$\log\left(\frac{p}{1-p}\right)∼m+h\;\text{(for relative trait values)}$$

We repeated the above analyses on species’ functional traits and natural distribution status of being close to (≤20 m) or far away from (>20 m) point count stations. We plotted the kernel density curves of body mass and foraging height among species distributed close to and far away from point count stations, displaying the trait positions of the five species producing the mobbing calls. We additionally conducted the hierarchical model analysis described above to test how these traits predicted species’ tendency of being close to point count stations, again using both raw and relative trait values. We conducted all analyses in R 3.2.3 (R Core Team 2015).

## 3 Results

Our point count and mist‐netting surveys detected a total of 158 resident nonraptorial bird species at the four study sites, while mobbing playback trials elicited the attraction response (defined in our study as the approach to within 15 m of the playback center) from a total of 71 species (Table S2). Responding species spanned 22 of the 33 families detected, were predominantly members of the order Passeriformes, but also included members of the orders Cuculiformes, Trogoniformes, Coraciiformes, and Piciformes. Thus, the component of the bird community that utilized the mobbing calls provided in our study accounted for ~45% of the bird community detected in our surveys in terms of the number of species, and covered a wide taxonomic breadth.

**Figure 2:**
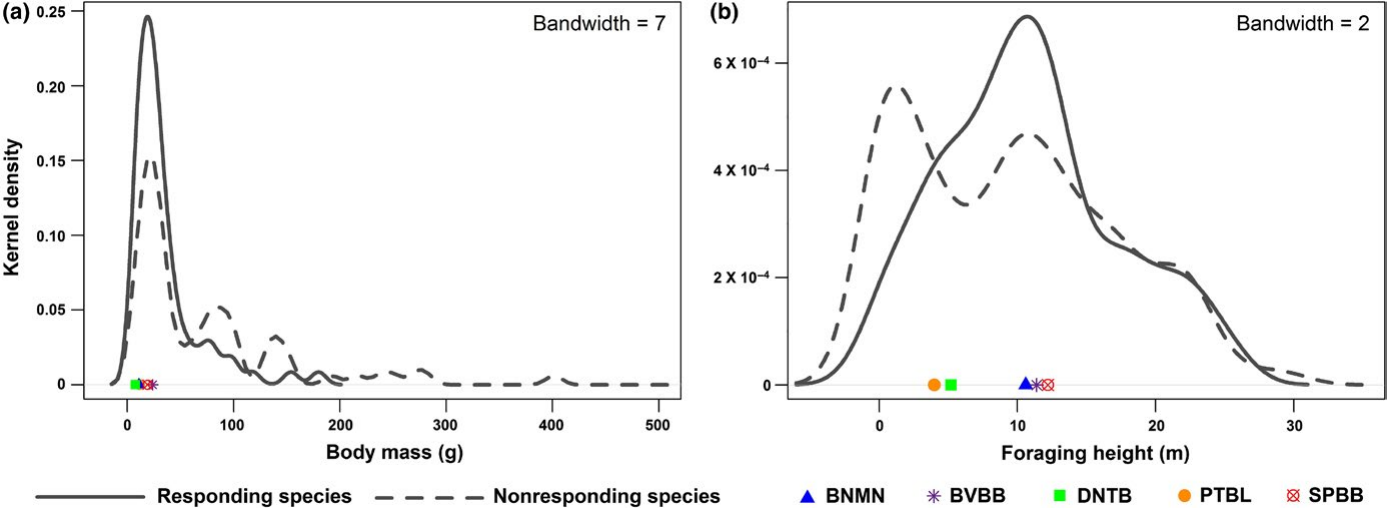
Kernel density curves of the risk‐determining functional traits of body mass (a) and foraging height (b) among species that responded to mobbing calls and those that did not. Solid lines represent species that responded to mobbing calls, while dotted lines represent those that did not. The locations of the five species that produced the mobbing calls on the curves are indicated by symbols in different shapes and colors. BNMN, black‐naped monarch; BVBB, buff‐vented bulbul; DNTB, dark‐necked tailorbird; PTBL, pin‐striped tit‐babbler; SPBB, spectacled bulbul

**Figure 3:**
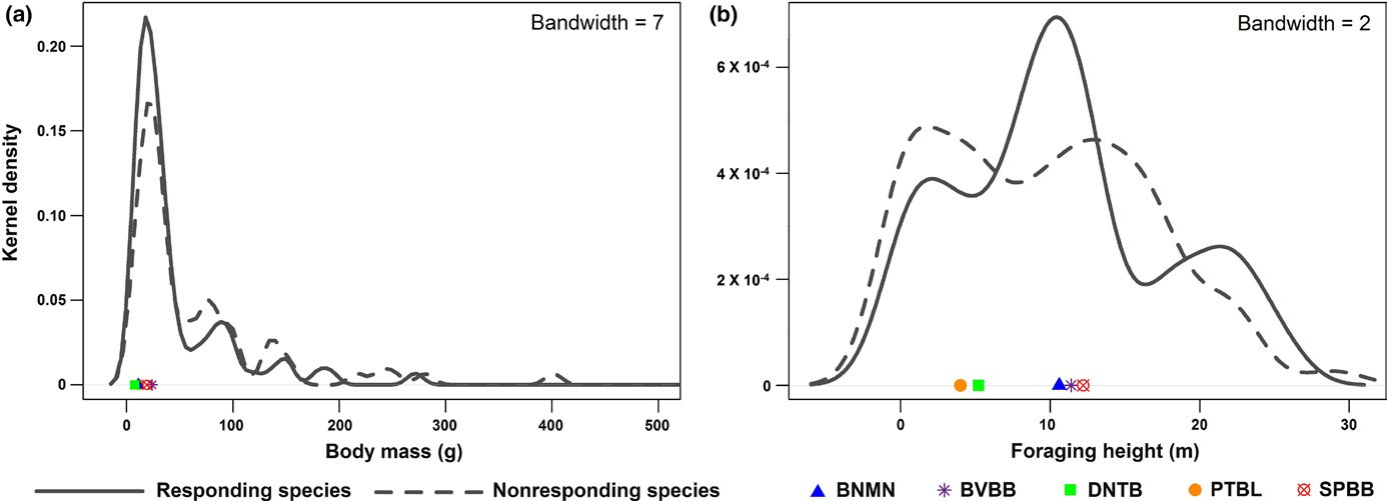
Kernel density curves of the risk‐determining functional traits of body mass (a) and foraging height (b) among species that were recorded close to (≤20 m) and far away from (>20 m) point count stations. Solid lines represent species that were recorded close to point count stations, while dotted lines represent those that were far away. The locations of the five species that produced the mobbing calls on the curves are indicated by symbols in different shapes and colors. BNMN, black‐naped monarch; BVBB, buff‐vented bulbul; DNTB, dark‐necked tailorbird; PTBL, pin‐striped tit‐babbler; SPBB, spectacled bulbul

The vast majority of responding species were of relatively small body mass (75% of responding species had body mass <38 g; Figure 2a) and intermediate foraging height between ground and canopy (>75% of responding species foraged between 3 and 18 m above ground; Figure 2b), as comparable to the traits of the five species that produced the mobbing calls. Compared with responding species, nonresponding species generally exhibited a larger body mass and lower or higher foraging height (Figure 2). Thus, responding species exhibited clustering in the trait space concerning body mass and foraging height that was largely around the position of the producers of mobbing calls. In comparison, species that were recorded to be close to point count stations did not exhibit clear patterns of clustering in trait space for either body mass (Figure 3a) or foraging height (Figure 3b), or separation in trait space from species not recorded to be close to point count stations (Figure 3), suggesting that the observed trait clustering of mobbing‐responsive species was not an artifact of these species incidentally occurring near the playback centers.

We found that species’ tendency to respond to mobbing calls was strongly predicted by their body mass and foraging height. According to AIC‐based model selection when using raw trait values, the best model contained the linear terms of body mass, and the quadratic and linear terms of foraging height (Table S4). Specifically, a species’ tendency to respond to mobbing calls was negatively correlated with body mass and exhibited a downward‐facing parabolic relationship with foraging height that peaked at the intermediate height of 14.03 m, just above the “mid‐high” stratum and coinciding with the foraging height of *Otus* owls in Sundaic forests (del Hoyo et al., 1992–2013; Table 1). Our analysis using relative trait values suggested that in terms of body mass and foraging height, the more similar a species was to the species producing the mobbing calls, the more likely it was to respond to the calls (Table 1). Therefore, risk‐related functional traits strongly predicted species’ tendency to use the social information of mobbing calls. In comparison, we did not find evidence of a strong relationship between functional traits and species’ tendency to be distributed close to point count stations (Table 2). Analysis using raw trait values suggested that similar to the attraction to mobbing calls, smaller birds tended to have stronger tendencies to be distributed close to point count stations, but this relationship was very weak (β = −0.36 compared with β = −9.02 in the case of attraction to mobbing calls); foraging height was not found to have a significant effect (Table 2). For analysis using relative trait values, neither body mass or foraging height was found to have a significant relationship with species’ tendency to be distributed close to point count stations (Table 2). Therefore, the strong relationship we found between species’ functional traits and their tendencies to respond to mobbing calls was unlikely to be an artifact of their natural distribution patterns.

**Table 1:** Relationship between functional traits and species’ tendency of responding to mobbing playbacks (on a logit scale)

Trait measure	Functional trait	β^a^	SE	95% CI	
Raw trait values	Body mass	−9.02	1.50	−11.96	−6.08
	Foraging height	0.54	0.13	0.29	0.79
	Foraging height^2^	−0.49^b^	0.11	−0.70	−0.27
Relative trait values	Body mass	−8.44	1.53	−11.44	−5.44
	Foraging height	−0.33	0.11	−0.55	−0.10

a Covariate values were calculated for variables that were centered and scaled.

b The back‐converted foraging height at which the parabolic relationship peaked was 14.03 m.

**Table 2:** Relationship between functional traits and species’ tendency of being recorded close to point count stations (on a logit scale)

Trait measure	Functional trait	β^a^	SE	95% CI	
Raw trait values^b^	Body mass	−0.36	0.18	−0.71	−0.02
	Foraging height	0.19	0.11	−0.02	0.40
Relative trait values	Body mass	−0.34	0.18	−0.69	0.002
	Foraging height	−0.01	0.11	−0.23	0.20

a Covariate values were calculated for variables that were centered and scaled.

b The model with the lowest AIC score had a polynomial term for body mass whose 95% CI included 0; we present here results from the model with the next lowest AIC (∆AIC = 0.13; Table S5), which differed from the best model only by not including the polynomial term.

## 4 Discussion

Combining community surveys with playback‐based behavioral manipulations, we show that nearly half (~45%) of the prey bird species at our study sites in the lowland rainforest of Sumatra were attracted to the heterospecific mobbing calls of five small‐bodied, mostly under‐ and/or mid‐storey bird species. Moreover, these species represented a nonrandom subset of the wider resident bird community; similar to the five species producing the mobbing calls used in our playbacks, responding species tended to be small‐bodied foragers of the under‐ and mid‐storey forest strata (Figures 2 and 3). Stronger tendencies to respond to the mobbing calls were found in smaller species and species that forage closer to the mid‐storey, and correspondingly in species sharing more similar traits with the species producing the mobbing calls (Tables 1 and 2). Body size and foraging space use are two of the most important functional traits determining prey vulnerability to predation risk, particularly with regard to the type of predators that pose risk for prey (Brose et al., 2006; Cohen et al., 1993; Klecka & Boukal, 2013; Lima, 1993). Our study thus demonstrates that in the rainforest bird community of lowland Sumatra, prey use of heterospecific mobbing calls as a form of risk‐related social information is closely related to their vulnerability to predation risk and that the subset of bird community that readily uses this information constitutes a risk‐sharing community that is susceptible to a similar suite of predators as the information producers (e.g., Forsman & Mönkkönen, 2001; Forsman et al., 2007).

Our results provide empirical support for the hypothesis that the reduction in future predation risk is likely a major benefit and evolutionary driver of the heterospecific attraction to mobbing calls (Magrath et al., 2015). Despite the well‐documented ubiquity of the heterospecific attraction to mobbing calls in animal communities and their presumed fitness benefits (Hurd, 1996; Schmidt et al., 2010; Seppänen et al., 2007; Sieving et al., 2004; Templeton & Greene, 2007), empirical evidence for such benefits has remained limited. While not directly testing the acquisition of such benefits, our findings suggest a reduction in future predation risk for species responding to heterospecific mobbing calls with attraction behaviors. By approaching mobbing calls, species that share predators (and functional traits defining their vulnerability) with information producers (mob callers; Klecka & Boukal, 2013) can acquire highly relevant information on risk situations that are important to their own survival. Such information can include precise identification of predator location and type (Altmann, 1956; Hetrick & Sieving, 2012) and the relative threat perceived (e.g., based on predator or signaler status; Bērziņš et al., 2010; Sieving, Hetrick, & Avery, 2010), and can in turn reduce current and future uncertainty with regard to prey decision making (Dall, Giraldeau, Olsson, McNamara, & Stephens, 2005). In forest habitats, most avian predators achieve predation by way of surprise attacks (Ekman, 1986; Ferguson‐Lees & Christie, 2001; del Hoyo et al., 1992–2013; Shultz, 2001). Approaching the scene of mobbing to collect information on the identity and status of a perched predator and potentially address it can almost certainly enable responding birds to minimize the risk of future predation (Caro, 2005; Dugatkin & Godin, 1992; Magrath et al., 2015).

While there are other functional traits relevant to prey interactions with predators (e.g., foraging technique and predator detection/escape strategy; Lima, 1993; Sridhar et al., 2012), we consider it appropriate to the ecological context of our study to use body mass and foraging height as key traits defining species’ vulnerability to predation risk. The behavioral response examined in our study was birds’ attraction to heterospecific mobbing calls to within 15 m of the playback center, under the context that we simulated of a perched predator being mobbed. On the part of the prey species, such attraction confers the benefit of increased information about the predator (e.g., its status, area of use; Dall et al., 2005; Seppänen et al., 2007), but typically does not involve the high risk and intense antipredator behaviors prey face most of the time with regard to ambush predators and surprise attacks, because the predator is already well located and unlikely to attack (Altmann, 1956). Therefore, the aspect of prey vulnerability most directly relevant to our study should concern the identity of predators the prey are vulnerable to, and therefore most likely to mob, rather than the modes of predator–prey interactions such as predator detection and escape, to which traits such as foraging technique and predator detection/escape strategy would have been more relevant (Lima, 1993; Sridhar et al., 2012). Functionally, body size fundamentally defines whether a species can be caught, subdued, and consumed by a given predator species (Brose et al., 2006; Cohen et al., 1993; Templeton et al., 2005), while foraging height determines the spatial overlap between prey and potential predators and thus prey exposure to and likelihood of encountering predators in forest ecosystems. These two traits thus represent the most relevant functional traits to the ecological context concerned in our study.

One important limitation in the interpretation of our findings is that because predation risk‐related acoustic materials are extremely limited for our study system, we were compelled to use the same recording of the mobbing calls throughout the study. We acknowledge that the resulting pseudoreplication in playback stimulus limits the scope of the inference we can draw from our data (Kroodsma, Byers, Goodale, Johnson, & Liu, 2001): We cannot ascertain that the same attraction patterns or intensities would hold for alternative versions of the mobbing calls, or avian mobbing calls in our study system in general. However, the mobbing calls we used were produced during what was described as a naturally occurring mobbing event by a highly regarded ornithologist familiar with the study system (B. van Balen, personal communication). In addition, the acoustic features of these calls—harsh, easily locatable, and rapidly repeating—are consistent with those of typical mobbing calls from forest birds in other study systems and, more importantly, those of the vocalization responses of birds attracted to the playback centers with characteristic mobbing behaviors according to our field observations. Thus, we consider it reasonable to expect that birds’ attraction responses we observed in our study should at least be informative, and potentially representative, of the potential responses to the general category of mobbing calls represented by the mobbing calls we used.

Two other caveats to our study design are also worth noting. First, our study used the simultaneous mobbing calls of five species that, despite sharing largely similar traits, spanned a range of trait values (particularly in terms of foraging height; Figure 2b). The use of multispecies mobbing calls was again imposed by the limited availability of acoustic materials for the study system. However, this design may have elicited the attraction response from a wider range of species and thus increased the variability of traits among responding species than would have been the case if mobbing calls from a single species were used. The increased trait variability may thus have weakened the strength of the detected relationship between species’ tendency to respond to mobbing calls and their traits. However, we note that the above concern may also not be an issue because mobbing events often quickly attract the visual and vocal responses from multiple species (Hurd, 1996; as was shown by our study). As such, even if mobbing calls from a single species were used as the initial stimuli, mobbing calls from other responding species would likely join quickly to render the actual mobbing stimuli to practically come from multiple species. Second, in constructing the two‐tiered hierarchical models to analyze the relationship between species’ tendency to respond to mobbing calls and their traits, for each given species, we assumed the same tendency across the four study sites that differed in forest degradation condition. Without *a priori* knowledge of how each species’ tendency to respond may differ across study sites, this assumption was necessary for constructing the hierarchical models. While this assumption may have ignored potential between‐site variability in a species’ tendency to respond to mobbing calls, the detection of significant relationships between species’ response tendency and their traits, despite not accounting for such potential variability, suggests that our observations are applicable across both intact and secondary forest sites (i.e., it makes our test conservative in nature). We note also that we did not quantify birds’ mobbing behavior here, which are distinct from birds’ attraction to mobbing calls and may well be sensitive to variation in vegetation structure caused by forest degradation (Hua & Sieving, 2016). In contrast, this study suggests that information to be gained by approaching a mobbing aggregation led by heterospecifics has value across study sites regardless of forest degradation status.

Our study demonstrates the existence and diversity of a risk‐sharing bird community in the lowland rainforest of Sumatra, where a large number of species spanning a large number of families readily use heterospecific social information in the form of mobbing calls. This finding for the comparatively lesser known tropical ecosystems of Asia (Goodale & Kotagama, 2008) adds to the growing evidence for the ubiquity of heterospecific information networks in animal communities (Magrath et al., 2015; Schmidt et al., 2010; Seppänen et al., 2007). More importantly, we provide empirical support that the reduction in predation risk is likely both a major benefit and evolutionary driving force for the formation of such risk‐related information networks (Dugatkin & Godin, 1992; Magrath et al., 2015; Martínez, Gomez, Ponciano, & Robinson, 2016). In human endeavors, social information is key to success and risk reduction (e.g., Morris & Hyun, 2002), but in animal systems this link is so far elusive. Further understanding of animal information networks will benefit from empirical studies that directly test the nature and extent of reduced predation risk resulting from birds’ use of risk‐related social information.

## Conflict of Interest

Authors claim no conflict of interest.

## Data Accessibility

All original data are provided in the Supporting information.
