# Supplementary material for: Functional traits determine heterospecific use of risk‐related social information in forest birds of tropical South‐East Asia
Source: Ecol Evol. 2016 Oct 29;6(23):8485–94. doi: 10.1002/ece3.2545 (PMC5167021; doi:10.1002/ece3.2545)
Supplement: Supplementary file 1 [file ECE3-6-8485-s001.docx]

**Supporting information**

**Title**

**Functional traits determine heterospecific use of risk-related social information in forest birds of tropical Southeast Asia**

**Authors**

Fangyuan Hua^1,2^, Ding Li Yong^3^, Muhammad Nazri Janra^4^, Liza M. Fitri^4^, Dewi Prawiradilaga^5^, Kathryn E. Sieving^6^

1. *State Key Laboratory of BioControl, College of Ecology and Evolution/School of Life Sciences, Sun Yat-sen University, Guangzhou, Guangdong 510275, China*
2. *Program in Science, Technology, and Environmental Policy, Woodrow Wilson School of Public and International Affairs, Princeton University, NJ 08544, U.S.A.*
3. *Fenner School of Environment and Society, the Australian National University, Canberra, Australia*
4. *Biology Department, Faculty of Mathematics and Natural Science, Andalas University, Padang, West Sumatra, Indonesia*
5. *Indonesian Institute of Technology (LIPI), Cibinong, West Java, Indonesia*
6. *Department of Wildlife Ecology and Conservation, College of Agriculture and Life Sciences, University of Florida, Gainesville, FL 32611, U.S.A.*

**Corresponding author**

Fangyuan Hua ([hua.fangyuan@gmail.com](mailto:fhua@princeton.edu))

**Supporting note**

Our pilot fieldwork in the study system indicated that the playback of the Sunda scops-owl territorial call was ineffective in eliciting the mobbing response from potential avian prey specie. This lack of response was confirmed by a number of ornithologists familiar with the Sundaland lowland rainforest (B. van Balen & F. Rheindt, *pers. comm.*), and is most likely the result of the species’ limited predation risk on avian prey species. Compared to similarly small-bodied owl species whose vocalizations regularly elicit the mobbing response from avian prey species, e.g. the collared owlet *Glaucidium brodiei* in Southeast Asia and the Eastern screech-owl *Megascops asio* in Eastern North America, the Sunda scops-owl has much more limited reliance on birds for its diet (its diet is noted to be mostly large invertebrates and occasionally small birds), and is also much less active during diurnal hours (del Hoyo, Elliott & Christie 1992-2013). As such, it may not be as highly relevant a predator to avian prey as in the case of the other species that regularly elicit mobbing from avian prey, thus attracting less attention from avian prey in the form of mobbing, which is costly in terms of the time, energy, and opportunity cost they spend addressing the predator (Hua & Sieving in press).

Reference

del Hoyo, J., Elliott, A. & Christie, D. (1992-2013) Handbook of the Birds of the World. Lynx Edicions.

Hua, F. & Sieving, K. E. In press. Understory avifauna exhibits altered mobbing behavior in tropical forest degraded by selective logging. *Oecologia*.

**Supporting figures**

Fig. S1. Sonogram of the mobbing call (a) and owl territorial call (b) used in the playback trials. Note that the resolution of the time axis for the mobbing call is twice as high as that for the owl territorial call in order to display its fast rate of notes.

**
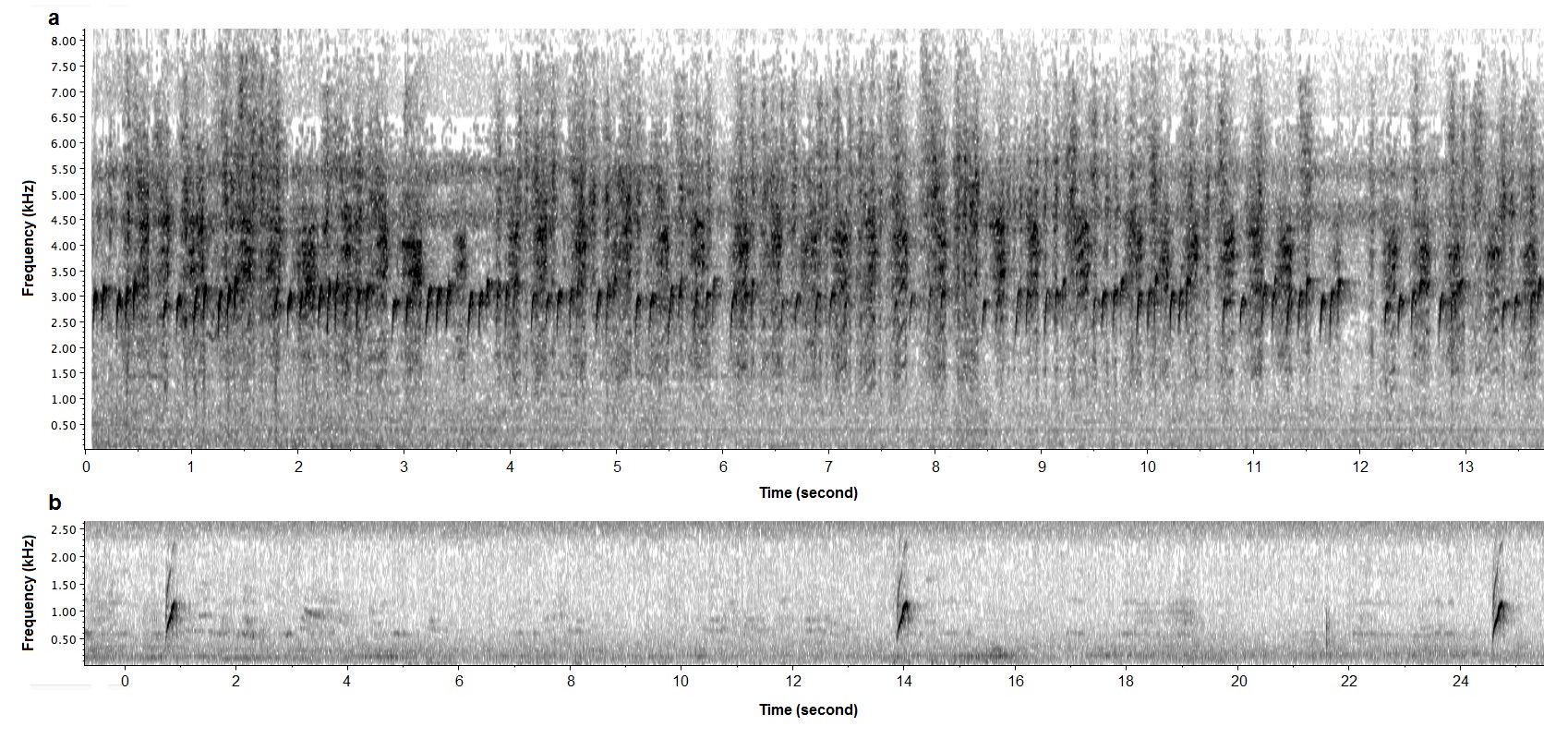
**

Fig. S2. Example layout of mist-nets during a full 9-day sampling period, at the DEG3 study site.


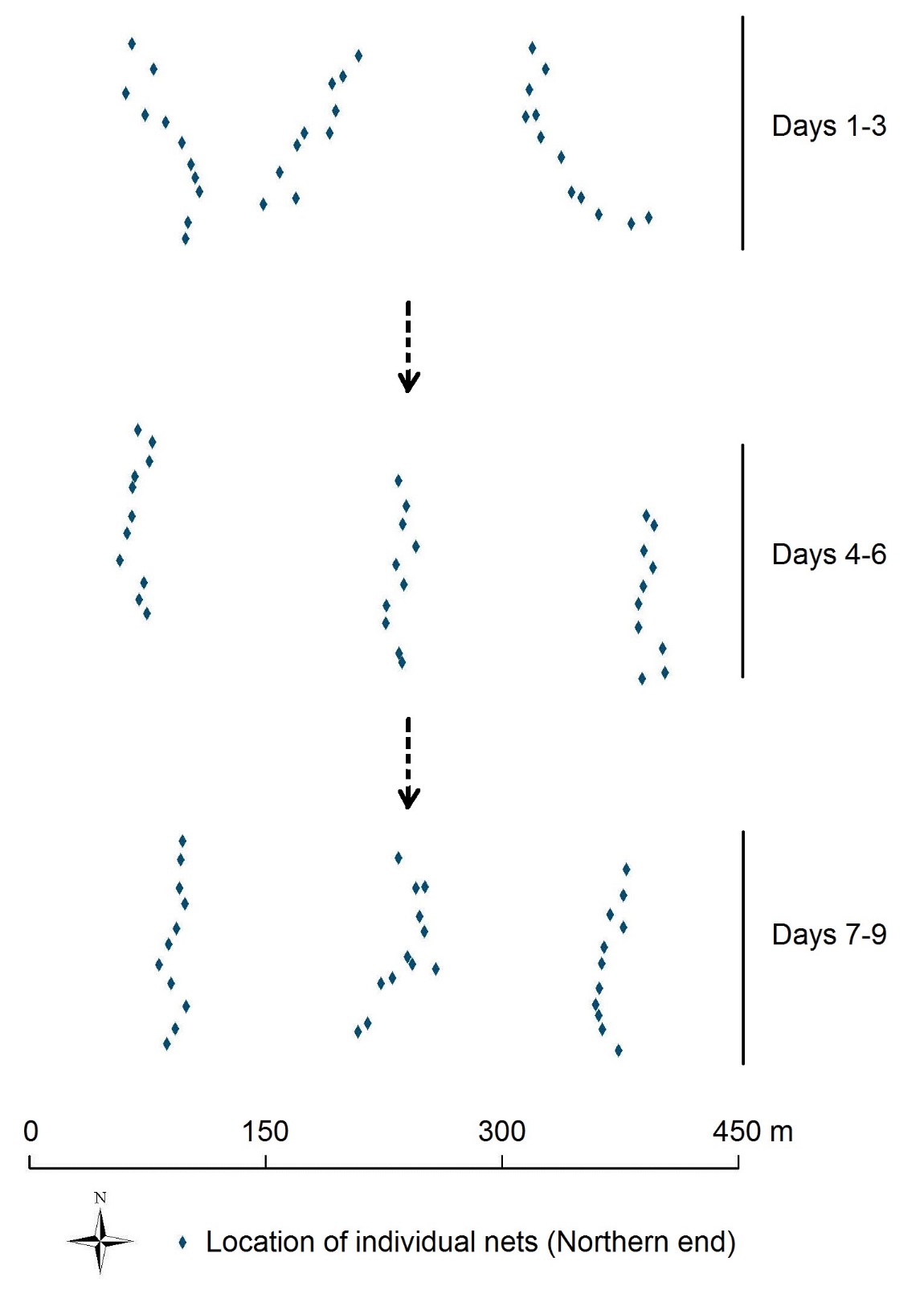


Fig. S3. Wooden model of the Sunda scops-owl in perched posture used as the visual stimulus in playback.

**
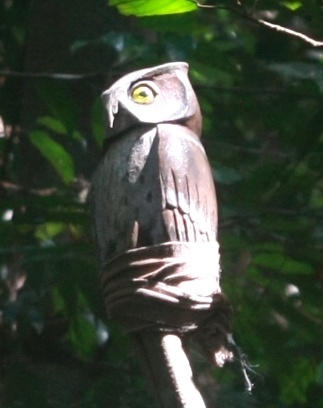
**

**Supporting tables**

Table S1. Body mass and foraging height of the five bird species that produced the mobbing calls used in the study. Taxonomy followed del Hoyo *et al.* (1992-2013).

| Species Latin name | Species English name | Body mass (g) | Foraging height (m)† |
| --- | --- | --- | --- |
|  |  |  |  |
| *Pycnonotus erythropthalmos* | Spectacled Bulbul | 19.2 | 12.21 |
| *Iole olivacea* | Buff-vented Bulbul | 24 | 11.4 |
| *Macronus gularis* | Pin-striped Tit-babbler | 11.83 | 4 |
| *Orthotomus atrogularis* | Dark-necked Tailorbird | 7.68 | 5.2 |
| *Hypothymis azurea* | Black-naped Monarch | 11.1 | 10.6 |

Note: † - Calculation of foraging height used the original (primary) set of height values (see Table S3).

Table S2. List of species that were detected at study sites by surveys (S) and that responded to mobbing calls (M), represented by “√”. Taxonomy followed del Hoyo *et al.* (1992-2013) unless noted otherwise.

| Species Latin name | Species English name | Body mass (g) | Foraging height (m)† | PRIM | | DEG1 | | DEG2 | | DEG3 | |
| --- | --- | --- | --- | --- | --- | --- | --- | --- | --- | --- | --- |
|  |  |  |  | S | M | S | M | S | M | S | M |
| *Rollulus rouloul* | Crested Partridge | 216.48 | 0 |  |  | √ |  |  |  |  |  |
| *Argusianus argus* | Great Argus | 1959.93 | 0.3 | √ |  | √ |  |  |  | √ |  |
| *Rallina fasciata* | Red-legged Crake | 100 | 0 | √ |  |  |  |  |  |  |  |
| *Treron curvirostra* | Thick-billed Green-pigeon | 134.45 | 9.74 | √ |  | √ |  | √ |  | √ |  |
| *Treron olax* | Little Green-pigeon | 77 | 12.8 | √ |  | √ |  | √ |  | √ |  |
| *Treron vernans* | Pink-necked Green-pigeon | 132 | 10.3 |  |  |  |  |  |  | √ |  |
| *Treron capellei* | Large Green-pigeon | 401.33 | 12.8 | √ |  |  |  |  |  |  |  |
| *Ramphiculus jambu* | Jambu Fruit-dove | 135 | 3.135 |  |  | √ |  |  |  |  |  |
| *Ducula aenea* | Green Imperial-pigeon | 545 | 17.6 | √ |  |  |  |  |  | √ |  |
| *Chalcophaps indica* | Grey-capped Emerald Dove | 136.54 | 2.95 | √ |  | √ |  | √ |  | √ |  |
| *Psittinus cyanurus* | Blue-rumped Parrot | 11.77 | 11.5 | √ |  | √ |  |  |  | √ |  |
| *Loriculus galgulus* | Blue-crowned Hanging-parrot | 28 | 15.2 |  |  | √ |  |  |  | √ |  |
| *Hierococcyx vagans* | Moustached Hawk-cuckoo | 57.7 | 7.5 |  |  |  |  |  |  | √ |  |
| *Cuculus micropterus* | Indian Cuckoo | 88.75 | 13.6 | √ |  |  |  | √ |  | √ |  |
| *Cacomantis sonneratii* | Banded Bay Cuckoo | 33.8 | 5.65 | √ |  | √ |  |  |  |  |  |
| *Cacomantis merulinus* | Plaintive Cuckoo | 25.3 | 15.2 |  |  |  |  | √ |  |  |  |
| *Chrysococcyx xanthorhynchus* | Violet Cuckoo | 22.37 | 7.8 |  |  | √ |  | √ |  | √ |  |
| *Surniculus lugubris* | Square-tailed Drongo-cuckoo | 29.7 | 17.6 | √ |  |  |  |  |  |  |  |
| *Phaenicophaeus sumatranus* | Chestnut-bellied Malkoha | 91.94 | 7.5 |  |  |  |  |  |  | √ |  |
| *Rhinortha chlorophaea* | Raffles’s Malkoha | 51.21 | 9.1 | √ | √ | √ |  | √ |  | √ |  |
| *Phaenicophaeus curvirostris* | Chestnut-breasted Malkoha | 154 | 17.6 | √ | √ |  |  |  |  |  |  |
| *Centropus rectunguis* | Short-toed Coucal | 238 | 12.8 |  |  | √ |  |  |  |  |  |
| *Centropus sinensis* | Greater Coucal | 280.7 | 0.45 |  |  | √ |  |  |  | √ |  |
| *Centropus bengalensis* | Lesser Coucal | 148.91 | 0.15 |  |  |  |  |  |  | √ |  |
| *Hemiprocne comata* | Whiskered Treeswift | 20.63 | 24 |  |  | √ |  |  |  |  |  |
| *Harpactes kasumba* | Red-naped Trogon | 96.89 | 7.9 | √ |  | √ |  | √ |  | √ |  |
| *Harpactes diardii* | Diard’s Trogon | 98.3 | 4.6 | √ |  | √ |  | √ |  | √ |  |
| *Harpactes duvaucelii* | Scarlet-rumped Trogon | 38.85 | 6.05 | √ | √ | √ | √ | √ | √ | √ | √ |
| *Alcedo meninting* | Blue-eared Kingfisher | 20.42 | 0.15 |  |  | √ |  | √ |  | √ |  |
| *Ceyx erithaca* | Oriental Dwarf-kingfisher | 17.79 | 0.45 | √ |  | √ |  |  |  | √ |  |
| *Ceyx rufidorsa*‡ | Rufous-backed Kingfisher | 17.79 | 0.45 | √ |  | √ |  | √ |  | √ |  |
| *Lacedo pulchella* | Banded Kingfisher | 47.27 | 1.75 | √ |  | √ |  |  |  | √ |  |
| *Halcyon smyrnensis* | White-breasted Kingfisher | 91.4 | 0 |  |  |  |  |  |  | √ |  |
| *Actenoides concretus* | Rufous-collared Kingfisher | 73.47 | 0 | √ |  | √ |  | √ |  | √ |  |
| *Merops philippinus* | Blue-tailed Bee-eater | 34 | 7.1 |  |  | √ |  |  |  | √ |  |
| *Nyctyornis amictus* | Red-bearded Bee-eater | 71.89 | 17.4 | √ |  | √ | √ | √ | √ | √ | √ |
| *Anorrhinus galeritus* | Bushy-crested Hornbill | 1172 | 6.45 | √ |  | √ |  | √ |  | √ |  |
| *Rhabdotorrhinus corrugatus* | Wrinkled Hornbill | 1590 | 16.5 | √ |  | √ |  |  |  | √ |  |
| *Rhyticeros undulatus* | Wreathed Hornbill | 2214.55 | 14 |  |  | √ |  |  |  |  |  |
| *Anthracoceros malayanus* | Black Hornbill | 1050 | 5.1 | √ |  | √ |  | √ |  | √ |  |
| *Buceros rhinoceros* | Rhinoceros Hornbill | 2371.58 | 14.8 | √ |  | √ |  | √ |  | √ |  |
| *Rhinoplax vigil* | Helmeted Hornbill | 2887.64 | 11.9 | √ |  | √ |  |  |  | √ |  |
| *Psilopogon chrysopogon* | Gold-whiskered Barbet | 151 | 17.6 | √ |  | √ |  | √ |  | √ |  |
| *Psilopogon rafflesii* | Red-crowned Barbet | 118 | 17.6 | √ |  | √ | √ | √ |  |  |  |
| *Psilopogon mystacophanos* | Red-throated Barbet | 77.4 | 4.75 | √ | √ | √ |  |  |  |  |  |
| *Psilopogon henricii* | Yellow-crowned Barbet | 74.1 | 17.6 |  |  | √ |  | √ |  | √ |  |
| *Psilopogon duvaucelii* | Black-eared Barbet | 33.3 | 14 | √ |  | √ |  | √ |  | √ |  |
| *Caloramphus hayii* | Malay Brown Barbet | 42.6 | 9.735 | √ |  | √ |  | √ | √ |  |  |
| *Sasia abnormis* | Rufous Piculet | 9.2 | 2.8 | √ | √ | √ | √ | √ |  | √ | √ |
| *Micropternus brachyurus* | Rufous Woodpecker | 85.94 | 8.85 | √ |  | √ |  | √ |  |  |  |
| *Dinopium rafflesii* | Olive-backed Woodpecker | 102 | 4.1 | √ |  |  |  |  |  |  |  |
| *Meiglyptes grammithorax* | Buff-rumped Woodpecker | 31.7 | 17.6 | √ |  | √ |  |  |  | √ |  |
| *Meiglyptes tukki* | Buff-necked Woodpecker | 53.1 | 9.735 | √ |  | √ |  |  |  | √ | √ |
| *Dryocopus javensis* | White-bellied Woodpecker | 271.4 | 4.75 |  |  | √ |  |  |  |  |  |
| *Blythipicus rubiginosus* | Maroon Woodpecker | 81.91 | 2.8 | √ |  | √ |  |  |  | √ |  |
| *Picus puniceus* | Crimson-winged Woodpecker | 79.1 | 3.135 | √ |  | √ |  | √ |  | √ |  |
| *Chrysophlegma humii* | Checker-throated Yellownape | 99.73 | 8 | √ |  | √ | √ |  |  | √ |  |
| *Chrysophlegma miniaceum* | Banded Woodpecker | 97.29 | 9.735 | √ |  |  |  |  |  | √ | √ |
| *Chrysocolaptes validus* | Orange-backed Woodpecker | 102 | 4.1 |  |  | √ |  |  |  | √ |  |
| *Corydon sumatranus* | Dusky Broadbill | 140 | 8 |  |  | √ |  | √ |  | √ |  |
| *Cymbirhynchus macrorhynchos* | Black-and-red Broadbill | 59.4 | 1.9 | √ |  | √ |  |  |  |  |  |
| *Eurylaimus javanicus* | Banded Broadbill | 78.2 | 3.95 | √ |  | √ |  | √ | √ | √ |  |
| *Eurylaimus ochromalus* | Black-and-yellow Broadbill | 33.3 | 3.45 | √ | √ | √ |  | √ |  | √ | √ |
| *Calyptomena viridis* | Green Broadbill | 58.5 | 3.95 | √ | √ | √ | √ | √ | √ | √ | √ |
| *Pitta granatina* | Garnet Pitta | 62 | 0.3 |  |  | √ |  | √ |  |  |  |
| *Pitta sordida* | Hooded Pitta | 64.5 | 0 | √ |  |  |  |  |  |  |  |
| *Pitta guajana* | Banded Pitta | 81.5 | 0 | √ |  | √ |  | √ |  | √ |  |
| *Hemipus hirundinaceus* | Black-winged Flycatcher-shrike | 10.2 | 20 | √ | √ | √ | √ | √ |  | √ | √ |
| *Tephrodornis virgatus* | Large Wood-shrike | 37.8 | 12 | √ |  | √ | √ | √ | √ |  |  |
| *Pericrocotus flammeus* | Scarlet Minivet | 23.3 | 20 | √ |  |  |  |  |  |  |  |
| *Aegithina viridissima* | Green Iora | 13.4 | 20 | √ | √ | √ | √ | √ | √ | √ | √ |
| *Aegithina tiphia* | Common Iora | 12 | 17.6 |  |  |  |  |  |  | √ |  |
| *Chloropsis cyanopogon* | Lesser Green Leafbird | 22 | 14 | √ | √ | √ | √ | √ | √ | √ | √ |
| *Chloropsis sonnerati* | Greater Green Leafbird | 45.8 | 14 |  |  | √ |  |  |  |  |  |
| *Chloropsis cochinchinensis* | Blue-winged Leafbird | 24.5 | 9.735 | √ | √ | √ | √ | √ |  | √ | √ |
| *Pycnonotus melanoleucos* | Black-and-white Bulbul | 31 | 9.735 |  |  | √ |  | √ |  |  |  |
| *Pycnonotus atriceps* | Black-headed Bulbul | 25.5 | 8.8 |  |  | √ | √ | √ | √ | √ | √ |
| *Pycnonotus dispar* | Ruby-throated Bulbul | 29.3 | 3.8 | √ | √ |  |  | √ | √ | √ | √ |
| *Pycnonotus cyaniventris* | Grey-bellied Bulbul | 22 | 17.6 |  |  | √ | √ | √ | √ | √ | √ |
| *Pycnonotus eutilotus* | Puff-backed Bulbul | 35.3 | 9.1 | √ |  | √ |  | √ |  | √ |  |
| *Pycnonotus plumosus* | Olive-winged Bulbul | 34.85 | 3.135 | √ |  | √ |  |  |  | √ |  |
| *Pycnonotus simplex* | Cream-vented Bulbul | 25.1 | 9.1 | √ | √ | √ | √ | √ | √ | √ | √ |
| *Pycnonotus brunneus* | Asian Red-eyed Bulbul | 28.8 | 7.9 | √ | √ | √ | √ | √ | √ | √ | √ |
| *Pycnonotus erythropthalmos* | Spectacled Bulbul | 19.2 | 12.21 | √ | √ | √ | √ | √ | √ | √ | √ |
| *Alophoixus finschii* | Finsch’s Bulbul | 24.2 | 9.1 |  |  | √ |  |  |  |  |  |
| *Alophoixus bres* | Grey-cheeked Bulbul | 35.31 | 4.75 | √ | √ | √ | √ | √ | √ | √ | √ |
| *Alophoixus phaeocephalus* | Yellow-bellied Bulbul | 32 | 4.75 | √ |  | √ | √ | √ | √ | √ | √ |
| *Tricholestes criniger* | Hairy-backed Bulbul | 17.1 | 6.7 | √ | √ | √ | √ | √ | √ | √ | √ |
| *Iole olivacea* | Buff-vented Bulbul | 24 | 11.4 | √ |  | √ |  | √ |  | √ |  |
| *Ixos malaccensis* | Streaked Bulbul | 37.3 | 18.6 | √ | √ | √ |  | √ | √ | √ |  |
| *Hemixos cinereus* | Cinereous Bulbul | 32.5 | 11.4 |  |  |  |  |  |  | √ |  |
| *Dicrurus aeneus* | Bronzed Drongo | 26 | 16.2 | √ | √ |  |  | √ | √ |  |  |
| *Dicrurus paradiseus* | Greater Racket-tailed Drongo | 85.82 | 10.15 | √ | √ | √ | √ | √ | √ | √ | √ |
| *Oriolus xanthonotus* | Dark-throated Oriole | 45 | 14.55 | √ | √ | √ | √ | √ | √ | √ |  |
| *Irena puella* | Asian Fairy-bluebird | 64.9 | 14 | √ | √ | √ |  | √ |  | √ | √ |
| *Platylophus galericulatus* | Crested Jay | 77.89 | 9.65 |  |  |  |  | √ |  |  |  |
| *Platysmurus leucopterus* | Black Magpie | 180 | 9.65 | √ | √ | √ | √ | √ |  | √ |  |
| *Corvus enca* | Slender-billed Crow | 251 | 4.45 |  |  | √ |  | √ |  | √ |  |
| *Sitta frontalis* | Velvet-fronted Nuthatch | 16.5 | 10.3 | √ |  | √ |  | √ |  | √ |  |
| *Pellorneum capistratum* | Black-capped Babbler | 25.1 | 0.3 | √ |  | √ |  | √ |  | √ |  |
| *Pellorneum buettikoferi* | Sumatra Babbler | 17.1 | 1.5 | √ |  |  |  |  |  |  |  |
| *Trichastoma rostratum* | White-chested Babbler | 17.8 | 0 | √ | √ | √ |  | √ |  | √ |  |
| *Trichastoma bicolor* | Ferruginous Babbler | 27.2 | 0.6 | √ |  | √ | √ | √ |  | √ |  |
| *Malacocincla malaccensis* | Short-tailed Babbler | 21.4 | 0.75 | √ |  | √ |  | √ |  | √ |  |
| *Malacocincla sepiarium* | Horsfield’s Babbler | 25.5 | 2.2 | √ |  |  |  |  |  | √ |  |
| *Malacopteron magnirostre* | Moustached Babbler | 19.34 | 8 | √ | √ | √ | √ | √ | √ | √ | √ |
| *Malacopteron affine* | Sooty-capped Babbler | 17.08 | 8 | √ |  | √ |  | √ |  | √ |  |
| *Malacopteron cinereum* | Scaly-crowned Babbler | 18.1 | 6.7 | √ | √ | √ | √ | √ | √ | √ | √ |
| *Malacopteron magnum* | Rufous-crowned Babbler | 27.2 | 6.7 | √ | √ | √ | √ | √ | √ | √ | √ |
| *Ophrydornis albogularis* | Grey-breasted Babbler | 16.5 | 1.5 |  |  | √ |  | √ | √ |  |  |
| *Pomatorhinus montanus* | Chestnut-backed Scimitar-babbler | 28.63 | 3.45 | √ | √ | √ | √ | √ | √ | √ | √ |
| *Kenopia striata* | Striped Wren-babbler | 19.9 | 0.75 |  |  | √ |  | √ | √ |  |  |
| *Stachyridopsis rufifrons* | Rufous-fronted Babbler | 10.6 | 2.8 |  |  | √ |  | √ |  | √ |  |
| *Stachyris poliocephala* | Grey-headed Babbler | 23.32 | 2.2 | √ |  | √ |  | √ |  | √ |  |
| *Stachyris maculata* | Chestnut-rumped Babbler | 29.2 | 6.7 | √ |  | √ | √ | √ | √ | √ | √ |
| *Stachyris leucotis* | White-necked Babbler | 23.7 | 0.75 |  |  | √ |  |  |  | √ |  |
| *Stachyris nigricollis* | Black-throated Babbler | 26.2 | 2.8 | √ | √ | √ |  | √ |  | √ |  |
| *Stachyris erythroptera* | Chestnut-winged Babbler | 12.6 | 8 | √ |  | √ |  | √ | √ | √ | √ |
| *Macronus gularis* | Pin-striped Tit-babbler | 11.83 | 4 | √ | √ | √ | √ | √ |  | √ | √ |
| *Macronus ptilosus* | Fluffy-backed Tit-babbler | 18 | 0.75 | √ | √ | √ |  | √ |  | √ |  |
| *Alcippe brunneicauda* | Brown Fulvetta | 14.3 | 6.7 | √ |  | √ | √ | √ | √ | √ | √ |
| *Eupetes macrocerus* | Rail-babbler | 101 | 0 | √ |  | √ |  | √ |  |  |  |
| *Trichixos pyrropygus* | Rufous-tailed Shama | 40.9 | 3.135 | √ |  | √ | √ | √ | √ | √ | √ |
| *Enicurus leschenaulti* | White-crowned Forktail | 33.8 | 0 | √ |  | √ |  |  |  | √ |  |
| *Orthotomus atrogularis* | Dark-necked Tailorbird | 7.68 | 5.2 | √ | √ | √ |  | √ | √ | √ | √ |
| *Orthotomus ruficeps* | Ashy Tailorbird | 8.1 | 9.735 | √ | √ |  |  |  |  |  |  |
| *Orthotomus sericeus* | Rufous-tailed Tailorbird | 10.8 | 0.3 | √ | √ | √ | √ | √ | √ | √ | √ |
| *Prinia familiaris* | Bar-winged Prinia | 9.68 | 7.375 |  |  |  |  |  |  | √ |  |
| *Rhinomyias olivaceus* | Fulvous-chested Jungle-flycatcher | 15.7 | 18.6 | √ |  | √ |  |  |  | √ |  |
| *Rhinomyias umbratilis* | Grey-chested Jungle-flycatcher | 18.2 | 7.8 | √ | √ | √ | √ | √ | √ | √ | √ |
| *Eumyias thalassinus* | Asian Verditer-flycatcher | 18.1 | 11.2 |  |  |  |  |  |  | √ |  |
| *Ficedula dumetoria* | Rufous-chested Flycatcher | 9.4 | 1.5 | √ |  |  |  |  |  |  |  |
| *Culicicapa ceylonensis* | Grey-headed Canary-flycatcher | 7.7 | 10.85 |  |  | √ |  |  |  | √ |  |
| *Cyornis unicolor* | Pale Blue-flycatcher | 21 | 13.2 | √ |  |  |  | √ |  | √ |  |
| *Cyornis caerulatus* | Large-billed Blue-flycatcher | 22.49 | 4.75 |  |  |  |  |  |  | √ | √ |
| *Rhipidura perlata* | Spotted Fantail | 13.4 | 7.2 |  |  | √ | √ | √ | √ |  |  |
| *Hypothymis azurea* | Black-naped Monarch | 11.1 | 10.6 | √ | √ | √ | √ | √ | √ | √ | √ |
| *Philentoma velata* | Maroon-breasted Philentoma | 26.1 | 8 |  |  | √ |  |  |  |  |  |
| *Philentoma pyrhoptera* | Rufous-winged Philentoma | 16.9 | 6.7 | √ | √ | √ | √ | √ | √ | √ | √ |
| *Terpsiphone paradisi* | Asian Paradise-flycatcher | 19.23 | 15.4 | √ | √ | √ |  |  |  | √ |  |
| *Gracula religiosa* | Common Hill Myna | 192 | 9.65 | √ |  | √ |  | √ |  | √ |  |
| *Anthreptes simplex* | Plain Sunbird | 8.93 | 7.25 | √ | √ | √ | √ | √ | √ | √ | √ |
| *Anthreptes malacensis* | Brown-throated Sunbird | 11.33 | 7.15 |  |  |  |  |  |  | √ |  |
| *Anthreptes rhodolaemus* | Red-throated Sunbird | 13.1 | 9.65 | √ | √ | √ | √ |  |  | √ | √ |
| *Chalcoparia singalensis* | Ruby-cheeked Sunbird | 8.2 | 10.3 | √ |  | √ | √ | √ | √ | √ | √ |
| *Hypogramma hypogrammicum* | Purple-naped Sunbird | 11.42 | 7.15 | √ | √ | √ | √ |  |  | √ | √ |
| *Leptocoma sperata* | Purple-throated Sunbird | 11.33 | 7.15 |  |  |  |  | √ |  |  |  |
| *Aethopyga siparaja* | Crimson Sunbird | 6.7 | 5.85 |  |  |  |  |  |  | √ |  |
| *Arachnothera longirostra* | Little Spiderhunter | 12.46 | 2.8 | √ | √ | √ | √ | √ | √ | √ | √ |
| *Arachnothera crassirostris* | Thick-billed Spiderhunter | 16 | 7.25 | √ |  |  |  |  |  |  |  |
| *Arachnothera robusta* | Long-billed Spiderhunter | 25.37 | 14 | √ | √ | √ | √ | √ | √ |  |  |
| *Arachnothera flavigaster* | Spectacled Spiderhunter | 38.4 | 11.6 | √ | √ | √ | √ | √ | √ |  |  |
| *Arachnothera chrysogenys* | Yellow-eared Spiderhunter | 23.2 | 14 |  |  | √ | √ | √ |  |  |  |
| *Arachnothera modesta* | Grey-breasted Spiderhunter | 26.4 | 12.7 | √ | √ | √ | √ | √ | √ | √ | √ |
| *Prionochilus thoracicus* | Scarlet-breasted Flowerpecker | 8.9 | 9.65 |  |  | √ |  | √ | √ |  |  |
| *Prionochilus maculatus* | Yellow-breasted Flowerpecker | 7.95 | 9 | √ | √ | √ | √ | √ | √ |  |  |
| *Prionochilus percussus* | Crimson-breasted Flowerpecker | 6 | 10.85 | √ | √ | √ | √ | √ | √ | √ | √ |
| *Dicaeum trigonostigma* | Orange-bellied Flowerpecker | 7.1 | 9.65 | √ | √ | √ |  | √ | √ | √ | √ |

Note: † - Calculation of foraging height used the original (primary) set of height values (see Table S3). ‡ - This species was formerly considered a separate species from its sister species *Ceyx erithaca*, and is now recognized by del Hoyo et al. (1992-2013) as the same species as *Ceyx erithaca*. In this study, we treated it as a standalone species.

Reference

del Hoyo, J., Elliott, A. & Christie, D. (1992-2013) Handbook of the Birds of the World. Lynx Edicions.

Table S3. List of alternative sets of height values, with height values of corresponding strata shown in meters.

| Alternative set | Ground level | Understory | Midhigh | Canopy | Aerial |
| --- | --- | --- | --- | --- | --- |
| 0† | 0 | 2 | 10 | 25 | 30 |
| 1 | 0 | 1.5 | 8 | 20 | 25 |
| 2 | 0 | 1 | 5 | 20 | 25 |
| 3 | 0 | 1.5 | 6 | 15 | 20 |
| 4 | 0 | 1 | 5 | 15 | 20 |

Note: † - Set 0 represents the original (primary) set of height values used in the main text.

Table S4. AIC scores of all competing models for the relationship between species’ functional traits (raw values, centered and scaled) and tendency of responding to mobbing calls (on a logit scale), for all alternative sets of height values.

| Alternative height set | Model structure | Number of variables | AIC |
| --- | --- | --- | --- |
| 0† | ~ body mass + foraging height + foraging height^2^ | 4 | 388.96 |
|  | ~ body mass + body mass^2^ + foraging height + foraging height^2^ | 5 | 390.70 |
|  | ~ body mass + foraging height | 3 | 407.57 |
|  | ~ body mass + body mass^2^ + foraging height | 4 | 409.31 |
|  | ~ body mass | 2 | 415.97 |
|  | ~ body mass + body mass^2^ | 3 | 417.70 |
|  | ~ foraging height + foraging height^2^ | 3 | 472.11 |
|  | ~ foraging height | 2 | 488.91 |
|  | ~ 1 | 1 | 493.50 |
| 1 | ~ body mass + foraging height + foraging height^2^ | 4 | 388.97 |
|  | ~ body mass + body mass^2^ + foraging height + foraging height^2^ | 5 | 390.71 |
|  | ~ body mass + foraging height | 3 | 407.71 |
|  | ~ body mass + body mass^2^ + foraging height | 4 | 409.45 |
|  | ~ body mass | 2 | 415.97 |
|  | ~ body mass + body mass^2^ | 3 | 417.70 |
|  | ~ foraging height + foraging height^2^ | 3 | 472.34 |
|  | ~ foraging height | 2 | 488.99 |
|  | ~ 1 | 1 | 493.50 |
| 2 | ~ body mass + foraging height + foraging height^2^ | 4 | 399.81 |
|  | ~ body mass + body mass^2^ + foraging height + foraging height^2^ | 5 | 401.55 |
|  | ~ body mass + foraging height | 3 | 412.22 |
|  | ~ body mass + body mass^2^ + foraging height | 4 | 413.95 |
|  | ~ body mass | 2 | 415.97 |
|  | ~ body mass + body mass^2^ | 3 | 417.70 |
|  | ~ foraging height + foraging height^2^ | 3 | 482.57 |
|  | ~ foraging height | 2 | 492.63 |
|  | ~ 1 | 1 | 493.50 |
| 3 | ~ body mass + foraging height + foraging height^2^ | 4 | 388.13 |
|  | ~ body mass + body mass^2^ + foraging height + foraging height^2^ | 5 | 389.88 |
|  | ~ body mass + foraging height | 3 | 407.59 |
|  | ~ body mass + body mass^2^ + foraging height | 4 | 409.33 |
|  | ~ body mass | 2 | 415.97 |
|  | ~ body mass + body mass^2^ | 3 | 417.70 |
|  | ~ foraging height + foraging height^2^ | 3 | 471.40 |
|  | ~ foraging height | 2 | 488.68 |
|  | ~ 1 | 1 | 493.50 |
| 4 | ~ body mass + foraging height + foraging height^2^ | 4 | 393.11 |
|  | ~ body mass + body mass^2^ + foraging height + foraging height^2^ | 5 | 394.85 |
|  | ~ body mass + foraging height | 3 | 409.92 |
|  | ~ body mass + body mass^2^ + foraging height | 4 | 411.66 |
|  | ~ body mass | 2 | 415.97 |
|  | ~ body mass + body mass^2^ | 3 | 417.70 |
|  | ~ foraging height + foraging height^2^ | 3 | 476.48 |
|  | ~ foraging height | 2 | 490.75 |
|  | ~ 1 | 1 | 493.50 |

Note: † - Set 0 represents the original (primary) set of height values used in the main text.

Table S5. AIC scores of all competing models for the relationship between species’ functional traits (raw values, centered and scaled) and tendency of being recorded close to point count stations (on a logit scale), for all alternative sets of height values.

| Alternative height set | Model structure | Number of variables | AIC |
| --- | --- | --- | --- |
| 0† | ~ body mass + body mass^2^ + foraging height | 4 | 371.08 |
|  | ~ body mass + foraging height | 3 | 371.21 |
|  | ~ body mass + body mass^2^ | 3 | 372.08 |
|  | ~ body mass | 2 | 372.28 |
|  | ~ body mass + body mass^2^ + foraging height + foraging height^2^ | 5 | 372.94 |
|  | ~ body mass + foraging height + foraging height^2^ | 4 | 373.05 |
|  | ~ foraging height | 2 | 375.94 |
|  | ~ 1 | 1 | 376.27 |
|  | ~ foraging height + foraging height^2^ | 3 | 377.76 |
| 1 | ~ body mass + body mass^2^ + foraging height | 4 | 371.08 |
|  | ~ body mass + foraging height | 3 | 371.21 |
|  | ~ body mass + body mass^2^ | 3 | 372.08 |
|  | ~ body mass | 2 | 372.28 |
|  | ~ body mass + body mass^2^ + foraging height + foraging height^2^ | 5 | 372.90 |
|  | ~ body mass + foraging height + foraging height^2^ | 4 | 373.01 |
|  | ~ foraging height | 2 | 375.93 |
|  | ~ 1 | 1 | 376.27 |
|  | ~ foraging height + foraging height^2^ | 3 | 377.72 |
| 2 | ~ body mass + body mass^2^ + foraging height | 4 | 371.64 |
|  | ~ body mass + foraging height | 3 | 371.77 |
|  | ~ body mass + body mass^2^ | 3 | 372.08 |
|  | ~ body mass | 2 | 372.28 |
|  | ~ body mass + body mass^2^ + foraging height + foraging height^2^ | 5 | 373.63 |
|  | ~ body mass + foraging height + foraging height^2^ | 4 | 373.75 |
|  | ~ 1 | 1 | 376.27 |
|  | ~ foraging height | 2 | 376.54 |
|  | ~ foraging height + foraging height^2^ | 3 | 378.53 |
| 3 | ~ body mass + body mass^2^ + foraging height | 4 | 371.06 |
|  | ~ body mass + foraging height | 3 | 371.18 |
|  | ~ body mass + body mass^2^ | 3 | 372.08 |
|  | ~ body mass | 2 | 372.28 |
|  | ~ body mass + body mass^2^ + foraging height + foraging height^2^ | 5 | 372.82 |
|  | ~ body mass + foraging height + foraging height^2^ | 4 | 372.91 |
|  | ~ foraging height | 2 | 375.87 |
|  | ~ 1 | 1 | 376.27 |
|  | ~ foraging height + foraging height^2^ | 3 | 377.61 |
| 4 | ~ body mass + body mass^2^ + foraging height | 4 | 371.31 |
|  | ~ body mass + foraging height | 3 | 371.44 |
|  | ~ body mass + body mass^2^ | 3 | 372.08 |
|  | ~ body mass | 2 | 372.28 |
|  | ~ body mass + body mass^2^ + foraging height + foraging height^2^ | 5 | 373.16 |
|  | ~ body mass + foraging height + foraging height^2^ | 4 | 373.27 |
|  | ~ 1 | 1 | 376.27 |
|  | ~ foraging height | 2 | 376.18 |
|  | ~ foraging height + foraging height^2^ | 3 | 378.01 |

Note: † - Set 0 represents the original (primary) set of height values used in the main text.

Table S6. Relationship between species’ functional traits (raw values, centered and scaled) and tendency of responding to mobbing playbacks (on a logit scale), for all alternative sets of height values excluding the original (primary) set.

| Alternative height set | Functional trait | β† | SE | 95% CI | |
| --- | --- | --- | --- | --- | --- |
| 1 | Body mass | -9.03 | 1.50 | -11.97 | -6.09 |
|  | Foraging stratum | 0.55 | 0.13 | 0.30 | 0.79 |
|  | Foraging stratum ^2^ | -0.49 | 0.11 | -0.70 | -0.27 |
| 2 | Body mass | -8.98 | 1.50 | -11.90 | -6.05 |
|  | Foraging stratum | 0.54 | 0.14 | 0.27 | 0.81 |
|  | Foraging stratum ^2^ | -0.41 | 0.11 | -0.63 | -0.19 |
| 3 | Body mass | -9.01 | 1.50 | -11.95 | -6.07 |
|  | Foraging stratum | 0.56 | 0.13 | 0.30 | 0.81 |
|  | Foraging stratum ^2^ | -0.49 | 0.11 | -0.71 | -0.27 |
| 4 | Body mass | -9.02 | 1.50 | -11.95 | -6.08 |
|  | Foraging stratum | 0.56 | 0.13 | 0.30 | 0.82 |
|  | Foraging stratum ^2^ | -0.46 | 0.11 | -0.68 | -0.24 |

Table S7. Relationship between species’ functional traits (relative values, centered and scaled) and tendency of responding to mobbing playbacks (on a logit scale), for all alternative sets of height values excluding the original (primary) set.

| Alternative height set | Functional trait | β† | SE | 95% CI | |
| --- | --- | --- | --- | --- | --- |
| 1 | Body mass | -8.45 | 1.53 | -11.45 | -5.44 |
|  | Foraging stratum | -0.32 | 0.11 | -0.55 | -0.10 |
| 2 | Body mass | -8.44 | 1.53 | -11.44 | -5.45 |
|  | Foraging stratum | -0.18 | 0.11 | -0.40 | 0.04 |
| 3 | Body mass | -8.43 | 1.53 | -11.44 | -5.43 |
|  | Foraging stratum | -0.33 | 0.12 | -0.56 | -0.11 |
| 4 | Body mass | -8.43 | 1.53 | -11.43 | -5.43 |
|  | Foraging stratum | -0.26 | 0.11 | -0.49 | -0.04 |

Table S8. Relationship between species’ functional traits (raw values, centered and scaled) and tendency of being recorded close to point count stations and functional traits (on a logit scale), for all alternative sets of height values excluding the original (primary) set†.

| Alternative height set | Functional trait | β‡ | SE | 95% CI | |
| --- | --- | --- | --- | --- | --- |
| 1 | Body mass | -0.36 | 0.18 | -0.71 | -0.02 |
|  | Foraging stratum | 0.19 | 0.11 | -0.02 | 0.40 |
| 2 | Body mass | -0.36 | 0.18 | -0.71 | -0.02 |
|  | Foraging stratum | 0.17 | 0.11 | -0.04 | 0.38 |
| 3 | Body mass | -0.36 | 0.18 | -0.71 | -0.02 |
|  | Foraging stratum | 0.19 | 0.11 | -0.02 | 0.40 |
| 4 | Body mass | -0.36 | 0.18 | -0.71 | -0.02 |
|  | Foraging stratum | 0.18 | 0.11 | -0.03 | 0.39 |

Note: † - The models with the lowest AIC score had a polynomial term for body mass whose 95% CI included 0; we present here results from the models with the next lowest AIC (∆AIC < 0.15 in all cases; Table S5), which differed from the best models only by not including the polynomial term. ‡ - Covariate values were calculated for variables that were centered and scaled.

Table S9. Relationship between species’ functional traits (relative values, centered and scaled) and tendency of responding to mobbing playbacks (on a logit scale), for all alternative sets of height values excluding the original (primary) set.

| Alternative height set | Functional trait | β† | SE | 95% CI | |
| --- | --- | --- | --- | --- | --- |
| 1 | Body mass | -0.34 | 0.18 | -0.69 | 0.001 |
|  | Foraging stratum | -0.01 | 0.11 | -0.22 | 0.20 |
| 2 | Body mass | -0.35 | 0.18 | -0.69 | 0.001 |
|  | Foraging stratum | 0.02 | 0.11 | -0.19 | 0.24 |
| 3 | Body mass | -0.34 | 0.18 | -0.69 | 0.002 |
|  | Foraging stratum | -0.01 | 0.11 | -0.23 | 0.20 |
| 4 | Body mass | -0.34 | 0.18 | -0.69 | 0.001 |
|  | Foraging stratum | 0.001 | 0.11 | -0.21 | 0.22 |
